# Disentangling the influences of mean body size and size structure on ecosystem functioning: an example of nutrient recycling by a non‐native crayfish

**DOI:** 10.1002/ece3.1852

**Published:** 2015-12-15

**Authors:** Keith J. Fritschie, Julian D. Olden

**Affiliations:** ^1^ School of Aquatic and Fishery SciencesUniversity of WashingtonSeattleWashington98105

**Keywords:** Biodiversity‐ecosystem function, Colorado River Basin, intraspecific variability, invasive, nonlinear averaging, *Orconectes virilis*

## Abstract

Body size is a fundamental functional trait that can be used to forecast individuals' responses to environmental change and their contribution to ecosystem functioning. However, information on the mean and variation of size distributions often confound one another when relating body size to aggregate functioning. Given that size‐based metrics are used as indicators of ecosystem status, it is important to identify the specific aspects of size distributions that mediate ecosystem functioning. Our goal was to simultaneously account for the mean, variance, and shape of size distributions when relating body size to aggregate ecosystem functioning. We take advantage of habitat‐specific differences in size distributions to estimate nutrient recycling by a non‐native crayfish using mean‐field and variance‐incorporating approaches. Crayfishes often substantially influence ecosystem functioning through their omnivorous role in aquatic food webs. As predicted from Jensen's inequality, considering only the mean body size of crayfish overestimated aggregate effects on ecosystem functioning. This bias declined with mean body size such that mean‐field and variance‐incorporating estimates of ecosystem functioning were similar for samples at mean body sizes >7.5 g. At low mean body size, mean‐field bias in ecosystem functioning mismatch predictions from Jensen's inequality, likely because of the increasing skewness of the size distribution. Our findings support the prediction that variance around the mean can alter the relationship between body size and ecosystem functioning, especially at low mean body size. However, methods to account for mean‐field bias performed poorly in samples with highly skewed distributions, indicating that changes in the shape of the distribution, in addition to the variance, may confound mean‐based estimates of ecosystem functioning. Given that many biological functions scale allometrically, explicitly defining and experimentally or statistically isolating the effects of the mean, variance, and shape of size distributions is necessary to begin generalizing relationships between animal body size and ecosystem functioning.

## Introduction

Trait‐based ecology holds considerable promise for forecasting the effects of global change on ecosystem functioning by scaling up the actions of individuals independent of their taxonomy (Luck et al. 2012). Across animal taxa, body size has long been considered a useful trait in determining species' responses to the environment, their ecological relationships, and their roles in ecosystem processes (Peters 1983; Woodward et al. 2005). The metabolic theory of ecology provides a mechanistic basis for the scaling relationships between body size and many system properties (Brown et al. 2007), suggesting that body size may represent a universal trait to predict the effects of biological change on ecosystem functioning (Seguin et al. 2014).

Mean body size continues to be the primary currency by which studies have forecasted size‐dependent, ecosystem‐level impacts of extinctions, invasions, and compositional change (e.g., Solan et al. 2004; Larsen et al. 2005; McIntyre et al. 2007; Rudolf and Rasmussen 2013a; Seguin et al. 2014). However, individual variation (i.e., the frequency distribution of body size, herein size structure) across a population or community may have meaningful effects on ecosystem functioning that are masked by the averaging properties of mean trait values. Most ecosystem functions scale nonlinearly with body size following a power law function,

(1) $$y=\alpha x^{b}$$

where *y* is the per capita or population‐level functional rate of interest, *x* is body size, *b* is a scaling coefficient and *α* is a normalization constant. When *b* ≠ 1, the function is nonlinear such that such that small and large organisms exhibit different functional rates per unit size. Thus, size distributions that are represented by the same mean body size but vary in the relative proportion of different organism size classes may have varying effects on aggregate ecosystem functioning. This prediction is formulated mathematically as Jensen's inequality, where for a nonlinear function *f*(*x*) and distribution of *x* values with mean $\overset{¯}{x}$ and nonzero variance, the average of the function, $\bar{f(x)}$, does not equal the function of the average, $f(\overset{¯}{x})$ (Ruel and Ayres 1999).

Despite recent research incorporating size structure in size‐based investigations of ecosystem functioning (Dangles et al. 2012; Toscano and Griffen 2012; Norkko et al. 2013; Rudolf and Rasmussen 2013b), a perennial challenge involves the logistical constraints of experiments or natural field observations where size structure is often confounded with mean body size. For any function of interest that is predicted to scale allometrically (e.g., foraging rate, nutrient recycling, productivity), varying the distribution around the mean body size will deterministically alter the aggregate sum of that function at the population level following Jensen's inequality. Duursma and Robinson (2003) derived an approximation of the bias attributed to ignoring the variation around the mean for an allometric power law (i.e., tree stem mass ~ diameter at breast height) as

(2) $$\text{Estimated Bias}\;\left(\%\right)=\frac{1}{2}\left[b\left(b-1\right)\right]{\text{CV}}^{2}$$

where *b* is the scaling coefficient of the power law and CV is the coefficient of variation of the size distribution. Accordingly, scientists studying the relationship between body size and ecosystem functioning may bias their estimates of the mean body size effect if variability also changes with their experimental design. This bias may be important given that both mean body size and size structure are subject to natural and human‐induced variation across time and space, including ontogenetic shifts, phenotypic plasticity, climate change, and size‐selective harvesting. Paralleling recent calls to consider the importance of both inter‐ and intraspecific trait variability in community ecology (Violle et al. 2014), scientists now acknowledge the potential benefits of individual‐level data in understanding the dynamics and functioning of ecosystems (e.g., Trebilco et al. 2013). An important body of work has emerged that relates community‐level responses of size spectra (the distribution of biomass across size classes: a method that links individual‐level data to communities and ecosystems) to environmental change and ecosystem functioning (Dossena et al. 2012; O'Gorman et al. 2012). However, the translation of these results to the commonly used framework of trait (body size) means and variances remains unclear.

As one of the few studies to incorporate multiple aspects of size distributions in ecosystem functioning studies, Allgeier et al. (2014) found that both maximum body size and skewness of size distributions were significant parameters in statistical models of coral reef fish community nutrient recycling. Nutrient recycling is an important ecosystem function in which consumers can control resource dynamics by remineralizing nitrogen and phosphorus through excretion (Vanni 2002). Numerous studies have demonstrated that nutrient recycling can interact with traditional trophic controls of primary production in both experimental (Knoll et al. 2009; Kohler et al. 2011) and natural systems (Vanni et al. 2006), can rival atmospheric (Schindler et al. 2001) or watershed inputs of inorganic nutrients (Vanni et al. 2006), and can relieve and reverse nutrient limitation in some ecosystems (Allgeier et al. 2013; Atkinson et al. 2013).

Three decades of research demonstrate that, broadly, body size is important to nutrient recycling. Individual body size explains most variation in per capita nutrient excretion rates (Sereda et al. 2008; Allgeier et al. 2015), although significant interspecific variability in this relationship may exist (Villeger et al. 2012; Allgeier et al. 2015). In aquatic systems, heterogeneous distributions of total consumer biomass can generate biogeochemical hotspots and hot moments – areas and times of elevated biogeochemical reaction rates (e.g., McClain et al. 2003; McIntyre et al. 2008; Atkinson et al. 2013). Species extinctions ordered by maximum body size had a larger effect on nutrient recycling than random extinction scenarios when population energy was conserved (McIntyre et al. 2007). Likewise, Hall et al. (2007) demonstrated that shifts in size structure affected lake nutrient recycling, and models containing both total fish biomass and abundance best explained aggregate nutrient recycling (Verant et al. 2007). Size is clearly important to nutrient recycling, but the relative contributions of total mass, mean body size, and size structure are difficult to compare in these studies and poorly understood in general.

Here we couple a field survey of a non‐native stream‐dwelling crayfish population (*Orconectes virilis* [Hagen, 1870]) in distinct habitat types with measures of individuals' rates of nutrient recycling to explore the relative contributions of mean body size and size structure to ecosystem functioning. Non‐native crayfishes are known to exert major impacts on the structure and functioning of the recipient system (Twardochleb et al. 2013). The few studies available suggest crayfishes recycle nutrients at high N:P ratios relative to other taxa (Evans‐White and Lamberti 2005; McManamay et al. 2011), likely because of the high P requirements of exoskeleton production for frequently molting crayfish (Habraken et al. 2015). Moreover, physical heterogeneity and ontogenetic preferences across longitudinal (i.e., riffle vs. run) and lateral (i.e., stream channel vs. stream bank) habitats lead to large variation in total mass, mean body size, and size structure within a crayfish population. Given the potential importance of crayfish to nutrient cycles and considerable local‐scale variability in their distribution, we asked (1) what is the relative contribution of mean body size and size structure to observed differences in nutrient recycling across habitat types after accounting for total biomass; and (2) does the shape of size structure variation around the mean alter the bias in nutrient recycling estimates? Specifically, we expected that (1) ontogenetic preferences would generate dissimilar body size distributions (mean, variance, and shape) across habitat types; (2) that variance‐induced bias in aggregate recycling estimates would be highest for habitat types with smaller mean body sizes; and (3) only the variance – not the shape – of the distribution would be important for predicting bias (following the bias approximation presented by Duursma and Robinson 2003).

## Methods

### System description

The Verde River is a large tributary (15,800 km^2^) in the Lower Colorado River Basin, Arizona, USA with a hydrologic regime controlled by a combination of perennial groundwater springs, cool wet winters (December ‐ March), and warm wet summer monsoons (July ‐ September) (Jaeger et al. 2014). The uppermost perennial section of the river (herein, ‘upper Verde’) runs 60 km from the run‐of‐river Sullivan Dam to its confluence with Sycamore Creek and maintained baseflows at 640 L/sec during our study period (April–June). The Verde River is one of two designated Wild and Scenic Rivers in Arizona and is a historical stronghold of several federal or state‐listed endemic species. However, a suite of human stressors and non‐native species serve as potential threats to this river ecosystem. The northern crayfish, *Orconectes virilis,* is native to the upper Midwest, USA and has been introduced widely across the Colorado River Basin (Martinez 2012), with common occurrences in the upper Verde River (Gibson et al. 2015). Given that no crayfish species are native to Arizona and that non‐native crayfishes have large effects on ecosystem functioning (Twardochleb et al. 2013), *O. virilis* may play a novel and important functional role in the Verde and other southwestern rivers (e.g., Moody and Sabo 2013).

### Individual nutrient recycling

We used standard incubations in the field to characterize nutrient excretion rates across a range of crayfish body sizes. We focus this analysis on ammonium (NH_4_‐N) recycling because (1) nitrogen is the historically limiting nutrient in Sonoran desert streams (Grimm and Fisher 1986); and (2) our results addressing the mean vs. size structure should hold for any ecosystem function like ammonium recycling that scales nonlinearly with body size. Results were qualitatively similar for phosphate (PO_4_‐P) recycling, and N:P recycling ratios of this *O. virilis* population were high relative to other aquatic organisms (~200) and similar to other crayfishes. Data for both nutrients are available online.

Crayfish were captured by hand and placed individually in clear polyethylene bags filled with 250–500 mL of deionized water. Bags were incubated for 45 min in stream margins to maintain ambient water temperature and minimize stress. After the incubation crayfish were removed and measured (length [cm] and mass [g]). Two water samples were drawn from the well‐mixed bag, filtered (Whatman GFF 0.45 *μ*m filter), and immediately frozen on dry ice for later analysis. Samples were analyzed for NH_4_‐N using the colorimetric salicylate–hypochlorite method (Bower and Holm‐Hansen 1980) on a flow injection analyzer (QuikChem 8000 Series, Lachat Instruments, Loveland, CO) at Northern Arizona University's Colorado Plateau Analytical Laboratory. N excretion rates were calculated for each sample after accounting for ambient nutrient concentrations in control bags. The per capita relationship between body size and nutrient recycling was modeled as a power law (eq. 1) by regressing N (NH_4_‐N umol/individual * h) on individual mass (g) using nonlinear least squares. Selecting individual crayfish from all habitat types was necessary to model nutrient recycling across the full range of crayfish body size in this system. This may have introduced bias in later aggregate calculations of nutrient recycling if individuals from different habitats recycled nutrients at different rates (e.g., because of habitat‐specific diet differences). However, even if present, this effect would not detract from the main goal of our study: to disentangle the effects of several aspects of size distributions on ecosystem functioning.

### Field sampling and characterizing size distributions

Stream habitat is longitudinally (riffle‐run sequences) and laterally (mid‐channel and stream bank) heterogeneous. Crayfish use this habitat differentially through their ontogeny to balance resource acquisition, predation risk, and life history requirements, generating spatial and temporal variation in local size distributions. We use this size variation as a natural experiment to examine the relative influences of mean body size and size structure on nutrient recycling.

Crayfish were surveyed in three riffles and three runs (channel units) at each of three 500–1000 m reaches in the upper Verde River. Within each channel unit we sampled crayfish in mid‐channel and bank areas (microhabitat units). In four randomly placed 1 m^2^ quadrats in each microhabitat unit we disturbed the substrate to a depth of 15 cm, as well as submerged vegetation, for 1 min and captured dislodged crayfish in a 2 m^2^, 500 um mesh seine held downstream. We visually scanned the quadrat to ensure that dislodged crayfish were directed into the seine. This method is not effective for individuals that dig deep burrows in the bank, but *O. virilis* is an infrequent tertiary burrower (Berrell and Chenoweth 1982) and visual evidence of burrowing during our sampling period was scarce. The total length (±1 mm) and mass (±0.1 g) of all crayfish >45 mm were recorded. Individuals of early molts (<45 mm) that reached high abundance were grouped in like size classes and characterized by the average measured lengths of at least 10 individuals. Across all samples, 3362 crayfish were captured. We used length and weight data from 443 individuals to define the length–weight relationship of the *O. virilis* population and estimate masses of individuals not directly weighed (Fig. S1).

### Characterizing size distributions

Habitat‐specific size descriptors were quantified by pooling all four quadrat samples, resulting in 9 × 2 channel units (riffle vs. run) × 2 microhabitat units (mid‐channel vs. bank): 36 samples. We withheld four samples with low total abundance (*N* < 5 per sample) from analyses. For each sample we calculated the mean body size (mass [grams]), coefficient of variation (CV) of body size, skewness of the body size distribution, and total biomass of all individuals. We compared these moments individually across habitat types using 2‐way analyses of variance (ANOVA), fitting ‘channel’ and ‘microhabitat’ as predictors.

We also summarized multiple aspects of size structure variation simultaneously using a multivariate ordination approach. The size structure of each sample was defined by its cumulative abundance profile (CAP): a stratification of the sample into nine discrete size classes (by mass) where the proportion of individuals greater than or equal to the lower limit of the size class is reported for each size class (De Cáceres et al. 2013). The size structure of samples (i.e., shape of CAPs) can be compared using unconstrained, multidimensional scaling ordination methods that summarize dominant gradients of variation in the CAPs (De Cáceres et al. 2013). Differences detected using this method are relatively robust to the number of size classes chosen a priori (De Cáceres et al. 2013). We calculated Bray–Curtis dissimilarity between samples’ CAPs using the *vegdistruct* function in the *vegclust* R package (De Cáceres et al. 2010) and ordinated this matrix into two dimensions using a principal coordinate analysis (PCoA). A permutational multivariate analysis of variance (Anderson 2001) and a test of multivariate dispersion were used to test for differences in the multivariate size structure between habitat types.

### Estimating aggregate nutrient recycling and quantifying bias

For each sample we both estimated and calculated true aggregate areal nutrient recycling rates (umol NH_4_‐N) to compare expected ecosystem functioning based on means with observed ecosystem functioning that incorporated size structure. We used a mean‐field approach to estimate aggregate recycling rates by applying the nutrient recycling model above to the mean body size of each sample and multiplying by the total sample count. Next, we repeated this process for every individual in the sample, rather than the mean body size, and summed across individuals to calculate the true aggregate recycling rate. Because we were interested in size structure effects and not total biomass effects, we standardized aggregate recycling rates by dividing each sample by its total biomass. This standardization by total biomass still preserves mass‐specific differences in nutrient recycling (i.e., 1 kg of large crayfish have different recycling rates than 1 kg of small crayfish). The difference between the estimated and true aggregate recycling rate is considered the true bias of each sample,

(3) $$\text{True Bias}\;\left(\%\right)=\frac{\text{True Recycling ‐ Mean‐field Recycling}}{\text{Mean‐field Recycling}}$$

a consequence of not considering size structure variation around the mean. We also estimated the expected bias of each sample using the derivation of Duursma and Robinson (2003) for power laws (eq. 2). Finally, we estimated a bias‐corrected aggregate recycling rate by subtracting the product of the mean‐field recycling estimate and the expected bias from the mean‐field recycling estimate,

(4) Bias‐corrected Recycling=Mean‐field Recycling−(Mean‐field Recycling×Expected Bias)

We took two steps to test our hypothesized relationships between size structure, aggregate recycling rates, and mean‐field bias. First we compared the distributions of true, mean‐field, and bias‐corrected estimates of aggregate nutrient recycling between habitat types. This allowed us to qualitatively assess the importance of including variance information to predict ecosystem functioning across crayfish groups with expected differences in size structure. Second, we plotted true and estimated bias against mean body size, CV, and skewness across all samples. Because we were interested in identifying general trends between these elements rather than defining quantitative relationships, we fit the data with loess smoothing and did not perform statistical analyses on regression models.

## Results

### Per capita nutrient recycling

**Figure 1:**
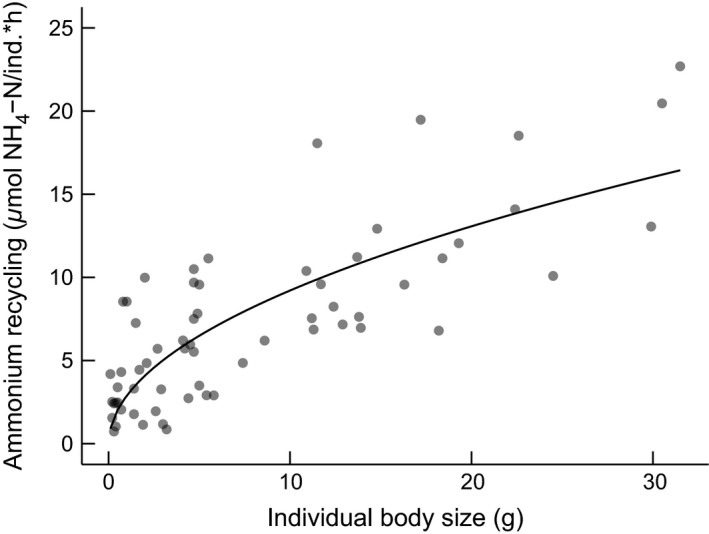
NH_4_‐N per capita recycling rates increased with individual body size following a decelerating power function (NH_4_‐N = 2.88 * Mass^0.50^). Each point is an individual crayfish captured independent of the field survey.

Individual body size was significantly correlated with NH_4_‐N recycling by crayfish (Fig. 1), with NH_4_‐N decelerating with increasing body size (parameter estimate ± standard error: *a *=* *2.88 ± 0.49, *b *=* *0.50 ± 0.06; *P *<* *0.001). This decelerating relationship between body size and excretion rate is common among aquatic consumers (e.g., Wen and Peters 1994; Sereda et al. 2008; Villeger et al. 2012), although this relationship can be isometric (e.g., Allgeier et al. 2015). Although details are not presented in full here, the N:P excretion ratio of *O. virilis* (mean = 200) was similar to other crayfish species (Lessard‐Pillon & McIntyre unpubl. data; Evans‐White and Lamberti 2005; McManamay et al. 2011) and high relative to other aquatic taxa.

### Crayfish size distributions across habitats

**Figure 2:**
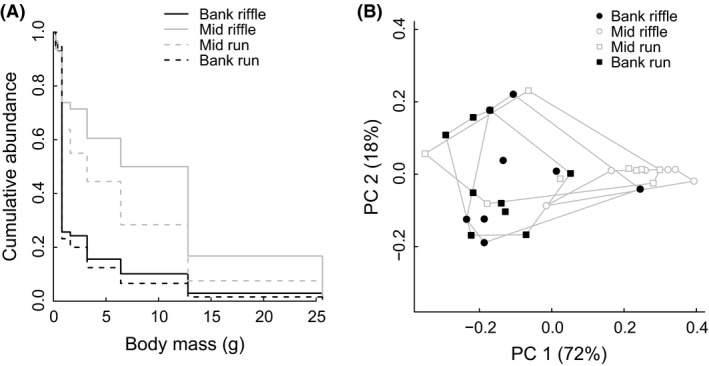
Differences in crayfish size structure between river habitat types. (A) The degree of overlap in samples’ cumulative abundance profiles (CAP) can be used to calculate a multivariate dissimilarity matrix between samples’ size structure. Average CAPs for each habitat type are presented here, with lateral habitat differences represented by color (black vs. gray) and longitudinal differences represented by line style (solid vs. dashed). (B) There were significant differences in size structure laterally through streams (black vs. gray) but not longitudinally (circles vs. squares).

Total biomass, mean body size, and size structure variability of crayfish varied differentially across stream habitat types (Table 1, Fig. 2, Fig. S2). Mean body size varied laterally (mean body size Mid = 5.5 g vs. Bank = 1.8 g: *F*_1,29_ = 25.9, *P *<* *0.001) but not longitudinally (Riffle = 4.3 g vs. Run = 3.0 g: *F*_1,29_ = 2.68, *P *=* *0.11). Total biomass varied both laterally (mean total biomass Mid = 31.1 g/m^2^ vs. Bank = 56.0 g/m^2^: *F*_1,29_ = 13.8, *P *<* *0.001) and longitudinally (Riffle = 31.2 g/m^2^ vs. Run = 56.0 g/m^2^: *F*_1,29_ = 9.1, *P *=* *0.005), but only the mid‐channel × riffle unit was significantly lower than the others when parsing apart habitat type differences with Tukey's HSD. CV also varied laterally (mean CV Mid = 1.1 vs. Bank = 1.8: *F*_1,29_ = 26.9, *P *<* *0.001) but not longitudinally (Riffle = 1.4 vs. Run = 1.5: *F*_1,29_ = 0.8, *P *=* *0.38). On average, distributions were right‐skewed (skewness > 0), and skewness varied laterally (mean skewness Mid = 1.6 vs. Bank = 3.6: *F*_1,29_ = 10.8, *P *=* *0.003) but not longitudinally (Riffle = 2.2 vs. Run = 3.0: *F*_1,29_ = 1.5, *P *=* *0.23).

**Table 1:** Habitat differences in *Orconectes virilis* total biomass and size structure metrics

Response and predictors	*F*	df	*P*	Habitat type means			
				Mid run	Mid riffle	Bank run	Bank riffle
Mean body size (g)				4.4 (ab)	6.6 (a)	1.5 (c)	2.0 (bc)
Channel (Riffle vs. Run)	2.7	1, 29	0.110				
Microhabitat (Bank vs. Mid)	25.9	1, 29	<0.001				
Total biomass (g/m^2^)				42.6 (ab)	19.6 (b)	69.3 (a)	42.7 (ab)
Channel (Riffle vs. Run)	9.1	1, 29	0.005				
Microhabitat (Bank vs. Mid)	13.8	1, 29	<0.001				
Coefficient of variation				1.1 (b)	1.0 (b)	1.9 (a)	1.72 (a)
Channel (Riffle vs. Run)	0.8	1, 29	0.380				
Microhabitat (Bank vs. Mid)	26.9	1, 29	<0.001				
Skewness				2.1 (ab)	1.2 (b)	3.9 (a)	3.3 (ab)
Channel (Riffle vs. Run)	1.5	1, 29	0.230				
Microhabitat (Bank vs. Mid)	10.8	1, 29	0.003				

Notes: Mean body size and biomass differences were analyzed with ANOVA on square root‐ transformed data. Interactive effects were not significant for any response so we report the results of the most parsimonious, main effects only models. Reported habitat type means are untransformed, and corresponding letters indicate significant differences according to Tukey's post hoc HSD (on square root transformed data).

Size structure *per se* differed laterally (mid‐channel vs. bank; perMANOVA *pseudo‐F*_1,31_ = 13.8, *R* ^2^ = 0.31, *P *=* *0.001) but not longitudinally (riffle vs. run; *pseudo‐F*_1,31_ = 1.3, *R* ^2^ = 0.03, *P *=* *0.24) (Fig. 2). Crayfish in stream banks were proportionally dominated by small‐bodied juveniles (<1 g) whereas large‐bodied juveniles and adults (>3 g) comprised a greater proportion of mid‐channel samples. Dispersion in ordination space (i.e., multivariate variability in CAPs) was not significantly different between habitat types (*F*_3,28_ = 0.61, *P *=* *0.61; Fig 2B).

### True and estimated aggregate nutrient recycling and bias

**Figure 3:**
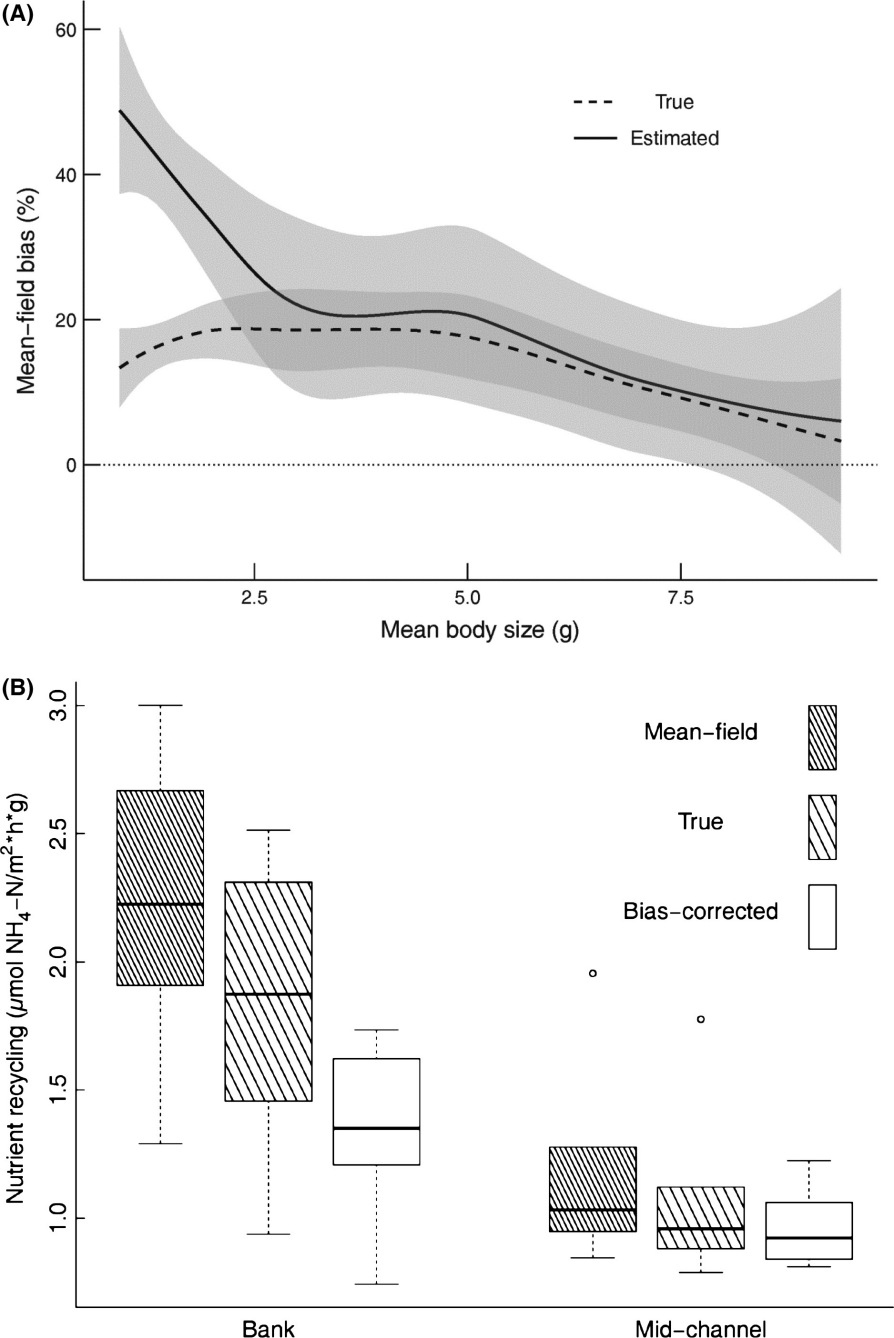
(A) The relationship between mean site body size and the true (dashed) vs estimated (solid) bias when using the mean‐field approach. Bias values near 0 indicate that the mean‐field approach and the true/estimated approach yield similar aggregate ecosystem functioning results. Positive bias values indicate that the mean‐field approach overestimates aggregate functioning. Gray shading indicates 95% confidence intervals around each loess‐smoothed model. (B) Differences in true, mean‐field, and estimated bias‐corrected aggregate nutrient recycling across habitat types. Aggregate recycling was standardized by the total biomass of the sample. For clarity this plot displays bank and mid‐channel differences for riffle units only. Results for runs were qualitatively similar.

**Figure 4:**
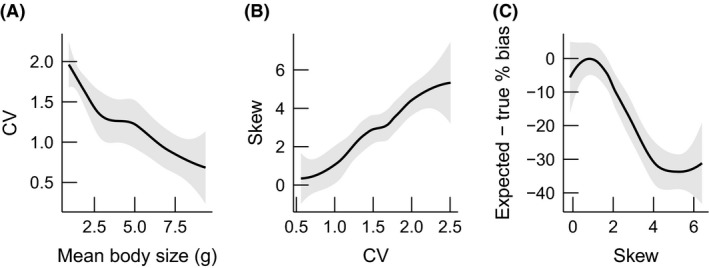
Relationships between (A) mean body size and CV, (B) CV and skew, and (C) skew and differences in true and estimated bias for NH_4_‐N per capita recycling rates across all samples. Gray shading indicates 95% confidence intervals around each loess‐smoothed model.

The mean‐field approach overestimated aggregate nutrient recycling (Fig. 3A) as predicted by Jensen's inequality for concave functions. Estimated bias ranged from 3.9 to 78.5% (mean ± SD: 30.0 ± 18.9) whereas true bias was lower but still positive on average (range: −4.3 to 28.0%; 14.8 ± 7.0). As expected, both true and estimated bias decreased with mean body size across all samples (Fig. 3A). For the bias estimation according to Duursma and Robinson (2003) (see eq. 2), this negative association is a direct consequence of a negative relationship between CV and mean body size (Fig. 4A). Decreasing importance of variance around the mean is also conceptually supported by a second derivative of the power law function that approaches zero with increasing mean body size (Fig. S3; sensu Inouye 2005). Consequently, differences in mean‐field, bias‐corrected, and true aggregate recycling rates were qualitatively greater in habitats with small mean body size (i.e., banks) than with large mean body size (mid‐channels) (Fig. 3B). In habitats with large mean body size, mean‐field approaches consistently overestimated true aggregate nutrient recycling, while correcting for bias following equation (2) consistently underestimated true functioning.

Although both true and estimated bias decreased with mean body size, these biases differed significantly at mean body sizes < ~2.5 g (Fig. 3A). Differences between true and estimated bias were as high as 52.6% in samples with low mean body size (1.6 g). At high CV values (i.e., low mean body sizes), size distributions were also very strongly positively skewed (Fig 4B). Increasing skewness was correlated with increasing differences between true and estimated bias (Fig. 4C), suggesting that the shape of the size distribution may exacerbate bias induced by changing CV.

## Discussion

Functional trait ecology has emerged as a powerful arena to predict species distributions and subsequent ecosystem functioning in natural communities (Violle et al. 2014). Intraspecific variability is expected to be important in relating traits to these population‐, community‐ and ecosystem‐level processes, but few studies explicitly account for the relative importance of mean body size and body size variation to animal‐mediated ecosystem functioning beyond inclusion in correlative statistical models (e.g., Allgeier et al. 2014). Consistent with predictions from Jensen's inequality, we found that individual variation around the mean body size reduced crayfish contributions to nutrient recycling and that the relative importance of variance (i.e., bias) decreased with increasing mean body size. However, bias predictions from Jensen's inequality diverged from reality as the shape of the size distribution became increasingly skewed.

Natural variation in nutrient recycling was generated by variable crayfish size distributions in the study system. Total biomass, mean body size, and size structure all varied laterally and longitudinally across unique habitat types. *O. virilis* in this lower portion of the Colorado River Basin reached substantially higher total biomass (mean = 41.8 vs. 15.8 g/m^2^), abundance (13.5 vs. 9.3 individuals/m^2^), and mean body size (3.1 vs. 2.0 g) than the same species invading the upper basin (Martinez 2012). After controlling for total biomass, we found that true aggregate ammonium recycling was highest among habitats with low mean body size and high CV – that is, banks (mean ± SD umol NH_4_‐N/m^2^ * h: 1.90 ± 0.44) vs. the mid‐channel (1.28 ± 0.51).

Comparing true recycling rates to the mean‐field approach (i.e., true bias) allowed us to then separate the importance of variance from the mean. Variance dampened aggregate recycling at a given mean (i.e., positive mean‐field bias), but the importance of variation decreased with increasing body size. This declining relationship can be attributed to the negative relationship between the CV and mean of body size in this study (Fig. 4A); following eq. 2, bias increases with the square of CV for power laws. Power law functions also become increasingly linear with increasing body size (i.e., second derivative approaches zero; Fig. S3), reducing the importance of variation around the mean (Ruel and Ayres 1999; Inouye 2005) independent of the negative empirical relationship between the mean and CV that we found. This latter reason for the decreasing importance of variance with increasing mean can be generalized to other ecosystem functions that scale with body size following power laws.

When mean body size >2.5 g, both true and estimated bias (eq. 2) generally agreed on the degree of mean‐field overestimation in functioning (i.e., overlapping confidence intervals, Fig. 3A). However, these bias calculations departed at low mean body size where true bias was significantly lower than that estimated from equation (2). We suspect that this difference is due to the increasing skewness of crayfish size distributions at high CV and low mean body size. Positive relationships between CV and skew are common for the log‐normal and Weibull‐like functions that often define body size frequency distributions, and in fact this relationship saturates such that there can be high variability in skewness within a very narrow range of CV for these particular distributions (Vargo et al. 2010). Higher order moments like skewness were not accounted for in the Taylor series expansion of Duursma and Robinson's (2003) bias estimate but are potentially important and could be incorporated with higher order expansion in future research.

Overall, we found distinct effects of the mean, variance, and shape of body size distributions on ecosystem functioning that were masked when considering only the mean. In the least, these higher order moments should be accounted for statistically when relating mean body size to ecosystem functioning. Beyond its role as a statistical nuisance, variation in body size distributions is interesting in its own right from ecological and evolutionary perspectives. Climate warming (Ohlberger 2013), sexual selection (Blanckenhorn 2000), size‐selective harvesting (Fenberg and Roy 2008), and intra‐ and interspecific interactions (Blanckenhorn 2000), among other processes, can act on body size within populations. The majority of studies on the phenotypic selection of body size focus on directional selection, with ~80% finding directional selection toward larger mean body size (Kingsolver and Pfennig 2004). However, the processes that may oppose this positive selection are understudied (Blanckenhorn 2000), and multiple processes acting in concert, or opposing selection on correlated traits, may generate temporally fluctuating signs of directional selection (Siepielski et al. 2009; Kingsolver and Diamond 2011) that influence both the mean and variance of trait distributions at time scales relevant to ecosystem functioning. Moreover, a smaller but substantial number of studies show that both stabilizing and disruptive selection act on body size (Kingsolver and Diamond 2011). These forms of selection directly influence the variance and shape of trait distributions without necessarily influencing the population mean. When biological and environmental forces act on body size in a way that decouples changes in the moments of size distributions, differentiating the roles of the mean, variance, and shape on ecosystem functioning may be of ecological, rather than just statistical, interest.

‘Functioning’ is broadly defined in ecology, and the effects of mean body size and size structure may follow different patterns for functions that are not distinct allometric rates of energy or material processing (e.g., prey community diversity and composition). Body size often relates to these functions according to discrete breaks or thresholds in body size rather than following a continuous allometric relationship. For example, body size alone could not predict basal ecosystem multifunctionality in aquatic invertebrate communities because individuals of different size classes interacted with lower trophic levels in fundamentally different, nonscalable ways (i.e., size‐dependent foraging preferences: Rudolf and Rasmussen 2013b). Moreover, changing animal behaviors may alter nonlinear body size‐function relationships, and deterministic predictions from Jensen's inequality may not perform realistically in such situations (Benedetti‐Cecchi 2005; Inouye 2005). How mean body size versus size structure affects these types of functions requires additional investigation. Benedetti‐Cecchi (2003) provides guidance on experimentally isolating the effects of the variance and mean of ecological processes, but we are unaware of a study that has extended this framework to body size‐ecosystem functioning relationships.

## Conclusion

Here we presented an example of how the mean and size structure can be confounded when explaining ecosystem functioning and highlight mathematical and experimental methods from related ecological fields to partition these effects (Ruel and Ayres 1999; Benedetti‐Cecchi 2003; Duursma and Robinson 2003; Inouye 2005). By doing so, we found that not accounting for variance can severely overestimate ecosystem functioning, especially at low mean body sizes, and that simultaneous changes to the shape of the size distribution confound straightforward methods to account for this mean‐field bias. Ecologists are increasingly interested in using size‐based indicators to gauge ecosystem status, and both mean body size and size structure‐based metrics have been proposed or are in operation (Shin et al. 2005; Petchey and Belgrano 2010). Yet the decision on which metric to use and how to set its reference condition is somewhat arbitrary and generally does not account for how size descriptors differentially reflect ecosystem functioning (Jennings & Dulvy 2005). We hope that explicitly defining and isolating the effects of the mean, variance, and shape of size distributions on ecosystem functioning in future basic research can lead to a better understanding of the size metrics appropriate for monitoring the functioning and health of ecosystems.

## Data accessibility

Data will be archived digitally for public access on FigShare: http://figshare.com/s/00eeadac584011e590ae06ec4bbcf141.

## Conflict of Interest

None declared.

## Supporting information


**Figure S1.** Length‐weight relationship of *O. virilis* measured in this study.
**Figure S2.** Habitat‐specific plots of crayfish size distributions.
**Figure S3.** Second derivative of per capita body size‐ammonium recycling model.
