# Supplementary material for: Disentangling the influences of mean body size and size structure on ecosystem functioning: an example of nutrient recycling by a non‐native crayfish
Source: Ecol Evol. 2015 Dec 15;6(1):159–69. doi: 10.1002/ece3.1852 (PMC4716502; doi:10.1002/ece3.1852)
Supplement: Supplementary file 1 — Figure S1. Length‐weight relationship of O. virilis measured in this study. Figure S2. Habitat‐specific plots of crayfish size distributions. Figure S3. Second derivative of per capita body size‐ammonium recycling model. [file ECE3-6-159-s001.pdf]

Supporting Information

Figure S1. Weight-length regression model for *Orconectes virilis* in the upper Verde River, Arizona, USA. Each point is an individual crayfish measured in the field. Methods following Froese (2005).

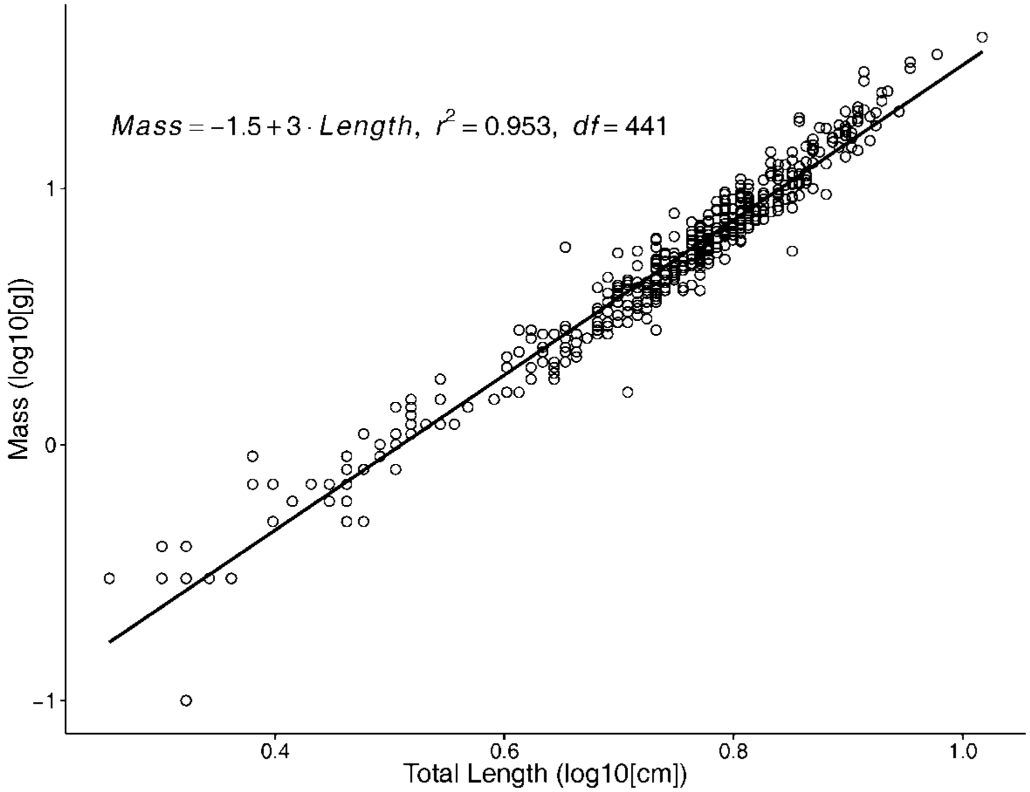

References

Froese, R. (2006) Cube law, condition factor and weight-length relationships: history, meta-analysis and recommendations. *Journal of Applied Ichthyology*, 22, 241-253.

Figure S2. Habitat-specific kernel density estimates of crayfish body size in this study pooled in Riffle (left column) and Run (right column) by bank (top row) and mid-channel (bottom row) habitat types. Data here are log-transformed for better visualization, though log-transforming skewed size distributions can either reveal or generate multimodality in the data (Wyszomirski 1992). We employed the multivariate method of De Cáceres, Legendre & He (2013) to quantify size distribution dissimilarities (alias Bray-Curtis) between plots to capture simultaneous differences in the mean, variance, and shape of distributions.

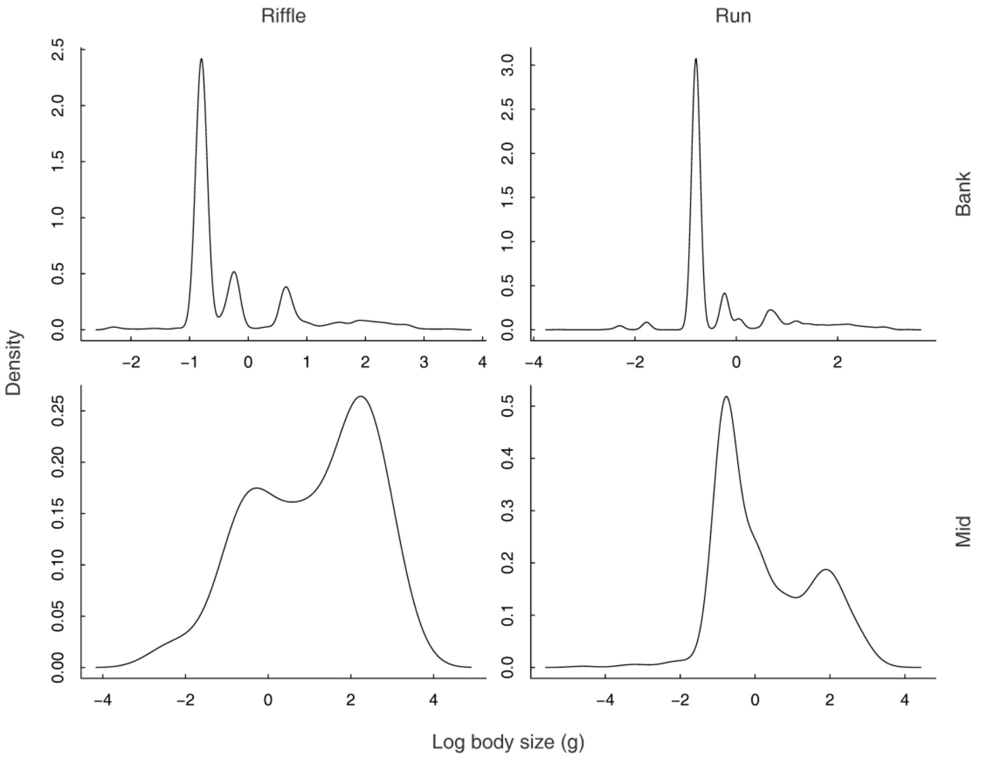

References

De Cáceres, M., Legendre, P. & He, F. (2013) Dissimilarity measurements and the size structure of ecological communities. *Methods in Ecology and Evolution*, 4, 1167-1177.

Wyszomirski, T. (1992) Detecting and displaying bimodality: kurtosis, skewness, and bimodalizable distributions. *Journal of Theoretical Biology*, 158, 109-128.

Figure S3. The second order derivative of the power law relationship between body size and per capita ammonium recycling rate (umol NH4-N/individual*hr). Following Ruel and Ayres (1999) and Inouye (2005), the second order derivative of the function provides an approximation of the importance of the variance of a process around the mean, with a non-zero variance indicating that variance will bias functioning estimated by the mean-field approach. This is a useful qualitative method to detect the potential importance of the variance, but does not provide an explicit estimate of mean-field bias.

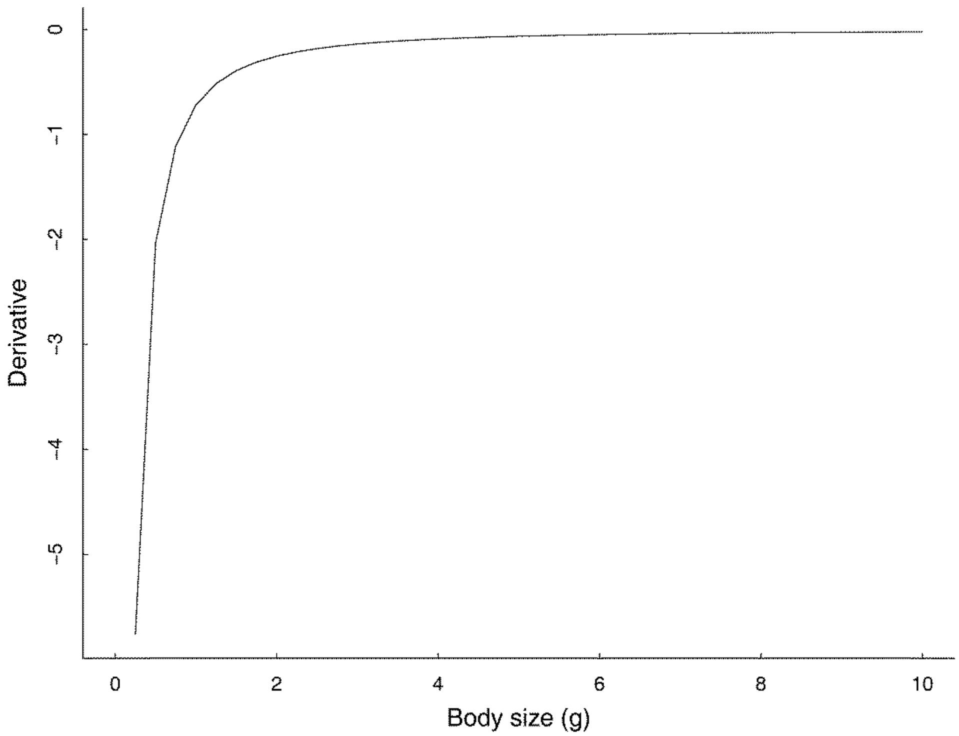

References

Inouye, B.D. (2005) The importance of the variance around the mean effect size of ecological processes: comment. *Ecology*, 86, 262-265.

Ruel, J.J. & Ayres, M.P. (1999) Jensen's inequality predicts effects of environmental variation. *Trends in Ecology and Evolution*, 14, 361-366.
